# Sampling and Characterization of Bioaerosols in Poultry Houses

**DOI:** 10.3390/microorganisms11082068

**Published:** 2023-08-11

**Authors:** Brooke L. Smith, Maria D. King

**Affiliations:** Aerosol Technology Laboratory, Biological & Agricultural Engineering Department, Texas A&M University, College Station, TX 77843, USA; blsmith5@tamu.edu

**Keywords:** antibiotic resistant bacteria, upwind, downwind, bioaerosol, confined animal feeding unit (CAFU)

## Abstract

Two poultry Confined Animal Feeding Units (CAFUs), “House A” and “House B”, were selected from the TAMU poultry facility for the study, and samples were collected over a five-day period. Bioaerosol sampling was conducted using a Wetted Wall Cyclone (WWC) bioaerosol collector at the two CAFU houses, in which House A housed approximately 720 broiler chickens and roosters, while House B remained unoccupied and served as a reference. Both houses consisted of 24 pens arranged on either side of a central walkway. Bacterial content analysis was conducted using microbial plating, real-time Polymerase Chain Reaction (PCR), and Fatty Acid Methyl Ester (FAME) analysis, while ambient temperature and relative humidity were also monitored. The concentrations of microorganisms in House A showed a highly dynamic range, ranging from 4000 to 60,000 colony forming units (CFU) per cubic meter of air. Second, the WWC samples contained approximately ten-fold more bacterial DNA than the filter samples, suggesting higher levels of viable cells captured by the WWC. Third, significant concentrations of pathogens, including *Salmonella*, *Staphylococcus*, and *Campylobacter*, were detected in the poultry facility. Lastly, the WWC system demonstrated effective functionality and continuous operation, even in the challenging sampling environment of the CAFU. The goal of this study was to characterize the resident population of microorganisms (pathogenic and non-pathogenic) present in the CAFUs and to evaluate the WWC’s performance in such an environment characterized by elevated temperature, high dust content, and feathers. This knowledge could then be used to improve understanding microorganism dynamics in CAFUs including the spread of bacterial infections between animals and from animals to humans that work in these facilities, as well as of the WWC performance in this type of environment (elevated temperature, high content of dust and feathers). A more comprehensive understanding can aid in improving the management of bacterial infections in these settings.

## 1. Introduction

The transmission of pathogenic bioaerosols and antibiotic resistance genes in poultry units is a concerning issue due to the health risk of birds, workers, and people in the surrounding areas of poultry units [1,2]. These bioaerosols are highly transmissible due to their aerodynamic properties characterized as their small diameter and lightweight [1,3]. These bioaerosols can include bacteria, fungi, viruses, and endotoxins; of the microbes in bioaerosols, more than 80% are bacteria [2,4].

Animal production has been identified as a significant source of bioaerosol concentration increase downwind of farming areas [1,5]. These bioaerosols can travel up to 1000 m downwind from the original source not only from outdoor sources but also from partially confined structures that can still spread bioaerosols to the ambient air due to winds [2]. Du et al. (2019) showed through experimentation and Computational Fluid Dynamics (CFD) modeling that higher concentrations of bioaerosols had a positive correlation to turbulence and disturbed airflow [3]. Factors influencing bioaerosol formation and transmission are convoluted and include age of the birds, stocking density, housing system, litter type, temperature relative humidity, air pollution, human activity, animal activity, land use, etc. Not only can these factors influence the formation of bioaerosols, but they can also affect the development of antibiotic resistance [6].

There also tends to be high bioaerosol concentrations around livestock and farms. The bacterial and fungal bioaerosols can range in size from 0.1 to 10 μm, which can be inhaled and potentially cause allergic reactions, cancer, and respiratory infections [7,8,9]. When exposed for long periods of time, workers or residents near poultry houses could develop lung dysfunction and severe systemic inflammatory responses occur, such as pneumonia, asthma, rhinitis, and various respiratory infections [9]. There have been multiple outbreaks at poultry facilities, such as in 2018, a multistate Psittacosis (caused by the bacterium *Chlamydia psittaci*) outbreak causing respiratory infections in employees [10]. Kasaeinasab et al. (2017) found that on average, 25% of workers in poultry environments suffered from cough, productive cough, breathlessness, phlegm, and wheezing, a significantly higher percentage compared to unexposed workers [11]. Higher concentrations of *Staphylococcus* were detected in bioaerosols that caused increased respiratory illnesses and symptoms in poultry facility workers. Endotoxins commonly released from pathogenic bacteria (*Staphylococcus*, *Campylobacter*, and *Salmonella*) during cell lysis can travel with larger aerosol particles and lead to immune functioning disorders and lung inflammation [9].

Numerous studies have highlighted the prevalence of Gram-positive and Gram-negative bacteria, such as Staphylococcus spp., including *Staphylococcus aureus*, *Escherichia coli, Streptococcus suis*, *Actinobacillus pleuropneumoniae*, *Micrococcus*, *Proteus*, *Pseudomonas*, and *Clostridia* in bioaerosol samples from poultry farming. Poultry facilities also contribute to fungal bioaerosols, most commonly *Aspergillus*, *Fusarium,* and *Penicillium* genera [12,13]. Previous research found that viruses could travel up to 50 km and Staphylococcae could travel up to 500 m [12]. While there are known risks, there are still gaps in research pertaining to the spread of pathogens from farm buildings and assessing the health risks to animals, residents, and farmers working in these environments.

Some common bacteria that cause illness including *Campylobacter*, *Staphylococcus,* and *Salmonella* are becoming increasingly antibiotic resistant. *Staphylococcus aureus* can cause skin and soft tissue infections, endocarditis, osteomyelitis, bacteremia, and lethal pneumonia. *S. aureus* has become increasingly resistant to antibiotics, especially methicillin-resistant *S. aureus* (MRSA), which is multi-drug resistant. Other drugs MRSA is resistant to include penicillins, cephalosporins, chloramphenicol, lincomycin, aminoglycosides, tetracyclines, macrolides, quinophthalones, sulfonamides, and rifampicin [14]. SEB, SEC, and SEA are genes found to produce enterotoxins causing foodborne illnesses [15]. *Salmonella enterica* causes foodborne infections and typhoid fever in humans and has developed resistance to multiple antibiotics, such as streptomycin, gentamicin, sulfadimethoxine, tetracycline, and the trimethoprim-sulfamethoxazole combination, negatively effecting food production and safety, especially in poultry [16]. A gene typically used for *Salmonella* identification *inv*A is a major factor in virulence of *Salmonella* [17]. *Campylobacter* is the cause of common diarrhea and specifically, *Campylobacter jejuni* is the most common cause of bacterial gastroenteritis. *Campylobacter* is becoming increasingly resistant to erythromycin, clarithromycin, azithromycin, and fluoroquinolones such as ciprofloxacin [18,19].

In this study, researchers used active sampling with a Wetted Wall Cyclone (WWC) bioaerosol collector. The WWC collects and concentrates ambient particles into a small volume of collection liquid, which increases the concentration of the particles in the sample by a factor of 10^6^ as compared to the ambient air. The WWC collector has been tested by the PIs in multiple settings including a laboratory flow cell where it effectively collected bioaerosols ranging in size from viruses (85 nm) to vegetative bacteria (up to 9.3 µm) [20]. Environmental sampling has been conducted in multiple indoor and outdoor locations for different time periods of hours to days. Hydrosols collected overnight (~50 mL) were concentrated to 100 µL using an InnovaPrep HSC-40. The WWC was chosen as the sampler due to its ability to preserve culturability and DNA integrity in the collected bacteria. In contrast to widely used impaction samplers for bioaerosol collection, the WWC maintains 100% culturability of *Bacillus atrophaeus* [aka *B. globigii*] spores [21]. King and McFarland (2012) were able to collect approximately 2 logs more of culturable *Escherichia coli* cells utilizing a WWC. This study also found that the DNA integrity was more similar to original stock suspensions of bacteria than other widely used bioaerosol collectors [20].

One method of isolation and identification of bacterial samples is Fatty Acid Methyl Ester (FAME) analysis. It has been commonly applied for bacteria using automated gas chromatography (GC) to hydrolyze fatty acids from phospholipids triacylglycerols, sterols, and any other lipids in the cell. The GC identifies the methyl esters formed to identify organisms [22,23].

This study aimed to characterize some of the resident population of microorganisms, including both pathogenic and non-pathogenic antibiotic resistant species, in Confined Animal Feeding Units (CAFUs) using the Wetted Wall Cyclone system (WWC). Two poultry CAFUs were chosen from the Texas A&M University (TAMU) poultry facility to be used in this study. The study lasted for five days (Monday–Friday). The collected samples were analyzed for bacterial content using microbial plating, real-time Polymerase Chain Reaction (PCR), and Fatty Acid Methyl Ester (FAME) analysis to establish a trend in concentration levels and identify the most frequently occurring bacteria. Ambient temperature and relative humidity were also monitored.

## 2. Materials and Methods

### 2.1. Sample Locations

Two CAFU “houses” of similar age, maintenance, and physical condition were selected. The first “House A” was home to a flock of approximately 720 broiler chickens and roosters. The second “House B” was empty at the time of the study and served as a testing “Reference”. This was used as a reference because it had the same layout with litter in the pens and was open to ambient air. The only difference was the presence of chickens in House A allowing the study to determine how the presence of chickens affects the bioaerosol composition.

**Figure 1:**
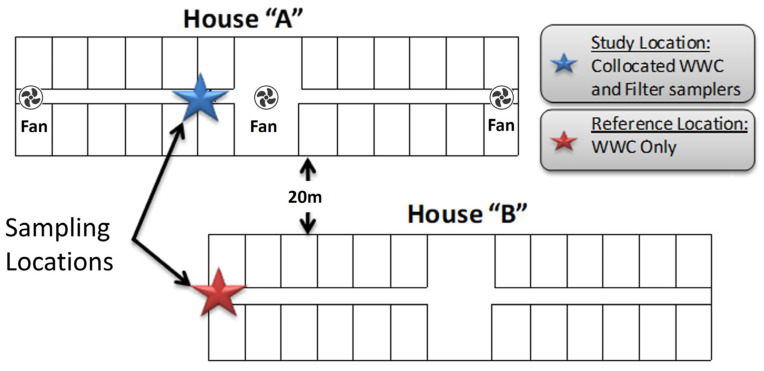
The layout of the two poultry Houses “A” occupied; “B” unoccupied at the TAMU Operated Poultry CAFU Facility. The location of the sampling devices is indicated by a blue (House A) or red (House B) star; the location of the three standing floor fans in House A is indicated by symbols.

The houses both contained 24 pens, each 10 ft × 10 ft in size and were arranged equally on both sides of a central walkway. Each pen was open to the walkway and had a 10 ft × 3 ft opening to the outside environment. The central walkway has two large ceiling fans and three large portable drum fans (ULINE, 9000 CFM, 1/3 HP, Pleasant Prairie, WI, USA) at the two ends with one additional fan in the middle. The concrete floors were hosed once during the five-day testing period of 13–17 June (Figure 1).

**Figure 2:**
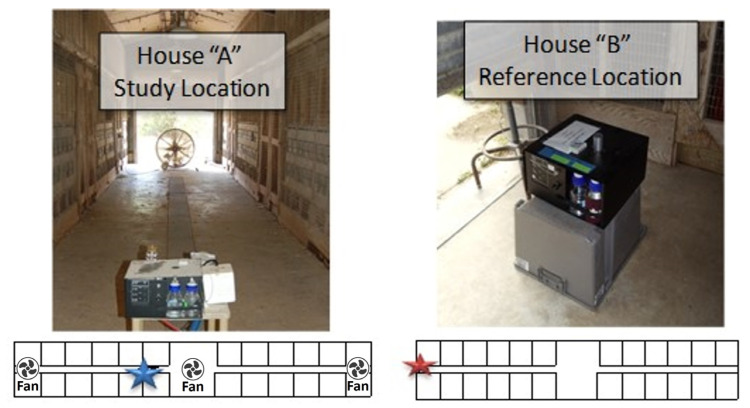
Location of the two samplers within Houses “A” and “B”. The location of the sampling devices is indicated by a blue (House A) or red (House B) star; the location of the three standing floor fans in House A is indicated by symbols.

The sampling location in House A was positioned a few feet from the main entrance of the central walkways. The Reference Sampler in House B was positioned in the open doorway with access to the indoor air as well as to ambient air (Figure 2) that would be most similar for both houses as the opening of House A is aligned with the collector in House B.

The sampling equipment used at the sampling location in House A consisted of a 100 L/min Wetted Wall Cyclone system [21]. As a collection liquid, 0.01% Tween-20 (VWR International, Radnor, PA, USA) was used at 200 µL/min inflow rate. The samples were collected in 50 mL Falcon tubes at 4 h intervals during the day and 16 h at night. As a Reference Sampler, 47 mm A/E Pall glass filters (Pall Corporation, Ann Arbor, MI, USA) were used at a flow rate of 100 L/min. The filters were immediately transported to the laboratory, where the collected material was resuspended in 5 mL of PBST (phosphate-buffered saline with 0.01% Triton, pH 7.4) (VWR International, Radnor, PA, USA).

The reference location in House B only had a 100 L/min Wetted Wall Cyclone system, which was operated in the same manner as at the study location in House A.

Samples were collected three times a day from both locations according to the following schedule: Morning samples were collected at 12:30 PM for a four-hour duration, Afternoon samples collected at 4:30 PM for a four-hour duration, and the Overnight sample were collected at 8:30 AM for a sixteen-hour duration. Continuous measurements of humidity and temperature were taken using HOBO Dataloggers (Onset, Bourne, MA, USA) during the collection of all bioaerosol samples, and the results were plotted to establish correlations between environmental conditions and bacterial concentrations.

All samples were transported directly to a biosafety level 2 (BSL2) laboratory for immediate analysis. The samples underwent three separate analysis methods: microbial plating, PCR, and FAME.

### 2.2. Plating and Analysis

Samples were amended with a supportive buffer, phosphate-buffered saline (PBS), before plating for appropriate dilutions. Researchers plated 100 µL of appropriate dilutions of the samples, which were plated on Tryptic Soy Agar (TSA) plates (Becton, Dickinson and Co., Sparks, MD, USA). The resulting colonies were counted and expressed as culturable colony forming units (CFU) after 18 h overnight incubation of the plates at 37 °C. There are limitations to culturability analysis using a bioaerosol collector because the only bacteria accounted for are those that are culturable. Only 1% of environmental bacteria collected in the environment maintain culturability [24], however recent studies indicate higher proportions across microbiome samples [25]. PCR was used to quantify the amount of bacteria in each sample whether culturable or non-culturable.

### 2.3. Quantitative Polymerase Chain Reaction (qPCR)

The PCR samples were also diluted and analyzed using universal 16S bacterial primers to quantify the total number of collected bacteria. The whole-cell quantitative PCR (qPCR) reaction mixture (10 µL total) contained the DNA template (3 µL of the collected samples), the forward and reverse primers (16S 1048 forward ‘5-GTGSTGCAYGGYTGTCGTCA’ and 1194 reverse ‘5-ACGTCRTCCMCACCTTCCTC’) (100 mM, 1 µL each), and Power SYBR Green PCR 2× Master Mix (5 µL, Life Technologies Ltd., Carlsbad, CA, USA) to amplify a 146 bp fragment [26]. Amplification and quantitation consisted of 15 min of denaturation at 95 °C, 40 cycles of annealing at 95 °C for 15 s, 60 °C for 60 s, and concluded with a holding temperature of 4 °C. For the standard curve, serial dilutions of fresh mid-log phase *E. coli* MG1655 bacteria (MG1655 (*E. coli* Genetic Resources at Yale CGSC, The Coli Genetic Stock Center, New Haven, NE, USA) with known colony forming units (CFUs) were used. Calculated total cell counts are expressed as Gene Copy Numbers (GCNs).

To quantify *Campylobacter,* the whole-cell quantitative PCR (qPCR) reaction mixture (10 µL total) contained the DNA template (3 µL of the collected samples), the forward and reverse primers (Cj oxidoreductase (oxred) forward 5′-CAA ATA AAG TTA GAG GTA GAA TGT and reverse 5′-GGA TAA GCA CTA GCT AGC TGA T) (100 mM, 1 µL each), and Power SYBR Green PCR 2× Master Mix (5 µL, Life Technologies Ltd.) to amplify a 160 bp fragment [27]. DNA was extracted from the samples using alkaline lysis [28].

To quantify *Salmonella,* the whole-cell quantitative PCR (qPCR) reaction mixture (10 µL total) contained the DNA template (3 µL of the collected samples), the forward and reverse primers (*inv*A 139 forward ‘5-GTGAATTATCGCCACGTTCGGGCA A’ and *inv*A 141 reverse ‘5TCATCGCACCGTCAAAGGAACC’) (100 mM, 1 µL each), and Power SYBR Green PCR 2× Master Mix (5 µL, Life Technologies Ltd.) to amplify a 284 bp fragment [29].

To quantify *Staphylococcus,* 3 targets were used, *sea*, *seb* and *sec* the whole-cell quantitative PCR (qPCR) reaction mixture (10 µL total) contained the DNA template (3 µL of the collected samples), the forward and reverse primers (*sea* forward ‘5- AAAGTGCCGATCAATTTATGGCTA’ and reverse ‘5- GTAATTAACCGAAGGTTCTGTAGA’) (100 mM, 1 µL each), and Power SYBR Green PCR 2× Master Mix (5 µL, Life Technologies Ltd.) to amplify a 219 bp fragment [30]. Secondly, the reaction mixture (10 µL total) contained the DNA template (3 µL of the collected samples), the forward and reverse primers (*seb* forward ‘5- TCGCATCAAACTGACAAACGA’ and reverse ‘5- CACTTTTTCTTTGTCGTAAGATAA’) (100 mM, 1 µL each), and Power SYBR Green PCR 2× Master Mix (5 µL, Life Technologies Ltd.) to amplify a 410 bp fragment [30]. Lastly, the reaction mixture (10 µL total) contained the DNA template (3 µL of the collected samples), the forward and reverse primers (*sec* forward ‘5- AACATTAGTGATAAAAAAGTGAAA’ and 1492 reverse ‘5- TTGTAAGTTCCCATTATCAAAGTG’) (100 mM, 1 µL each), and Power SYBR Green PCR 2× Master Mix (5 µL, Life Technologies Ltd.) to amplify a 234 bp fragment [30]. All primers were synthesized by Integrated DNA Technologies (IDTDNA Technologies, Coralville, IA, USA).

Amplification and quantitation consisted of 15 min of denaturation at 95 °C, 40 cycles of annealing at 95 °C for 15 s, 60 °C for 60 s, and concluded with a holding temperature of 4 °C. The primers specified were utilized for all bacteria (16S) and specific sequences to quantify species. The Cj oxred primer was used for *Campylobacter*, the *inv*A primer was used to quantify *Salmonella* presence in samples and the *sea*, *seb* and *sec* primers were used to quantify *Staphylococcus* in samples.

### 2.4. Fatty Acid Methyl Ester (FAME) Analysis

Fatty acids were extracted from samples by adding methanol. The fatty acid methyl esters were then analyzed by high resolution fused-silica capillary gas chromatography. Lipids have a conserved formation in bacteria, but the fatty acid content will vary in different samples [31]. Samples were harvested while in the late log phase of growth after 24 h incubation on TSBA media (VWR International, Radnor, PA, USA). After harvesting samples, a saponification reagent (sodium hydroxide, methanol, and DI water) (VWR International, Radnor, PA, USA) was added and the hydrochloric acid for methylation. For extraction, methyl tert-butyl ether (MTBE) and hexane (VWR International, Radnor, PA, USA) were added, and finally saturated sodium chloride (VWR International, Radnor, PA, USA) was added to wash the sample. FAME analysis was conducted using the (Agilent, Santa Clara, CA, USA) H5890 series GC. The unit was operated at 28 °C to detect species [32].

### 2.5. Statistical Analysis

Statistical significance was calculated to compare House A and House B and compare House A samples collected by the WWC and Reference Sampler. Statistical analysis was completed by using various *t*-tests to determine statistical significance using RStudio (RStudio 2022.12.0 + 353, Posit, PBC, Boston, MA, USA). The first null hypothesis stated that the mean culturability of House A would equal the mean culturability of House B. The second null hypothesis stated that the mean culturability of House A collected using the WWC would equal the mean culturability of House A using the Reference Sampler.

## 3. Results

### 3.1. Bacterial CFU Counts

**Figure 3:**
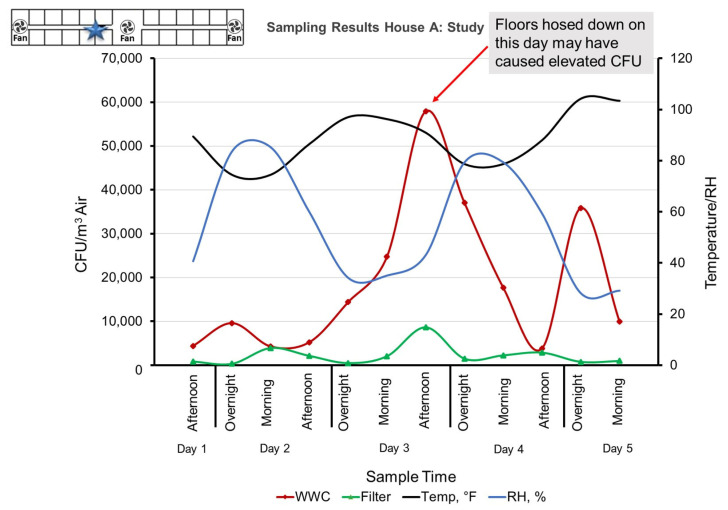
Sampling results of Study House A showing a highly dynamic range of bacterial concentrations as well as an order of magnitude increase in bacterial concentration from the filter collection to the WWC sampling (location indicated by a blue star). The Morning sample was collected at 12:30 PM for a four-h duration, Afternoon sample was collected at 4:30 PM for a four-h duration, and the Overnight sample was collected at 8:30 AM for a sixteen-h duration.

**Figure 4:**
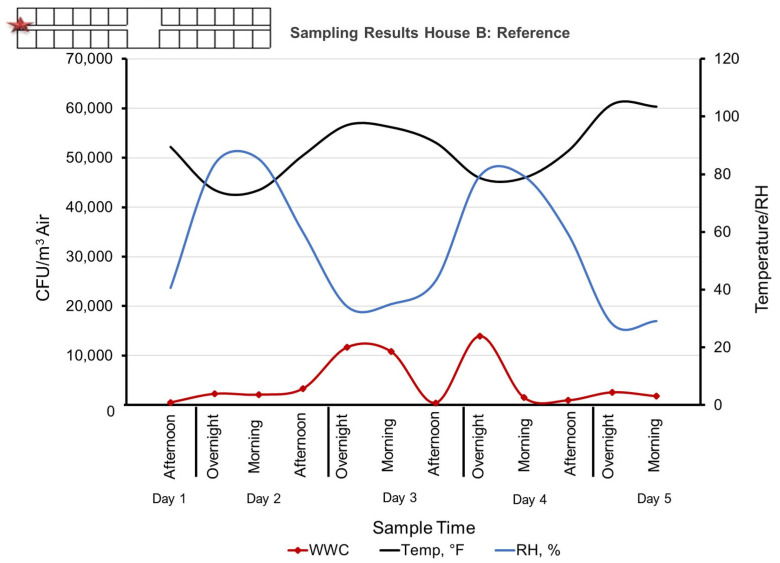
Sampling results of Reference House B showing a dynamic range of bacterial concentrations likely caused by contamination from House A, which is 20 m to the north. Sampling location is indicated by a red star. The Morning sample was collected at 12:30 PM for a four-h duration, Afternoon sample was collected at 4:30 PM for a four-h duration, and the Overnight sample was collected at 8:30 AM for a sixteen-h duration.

**Figure 5:**
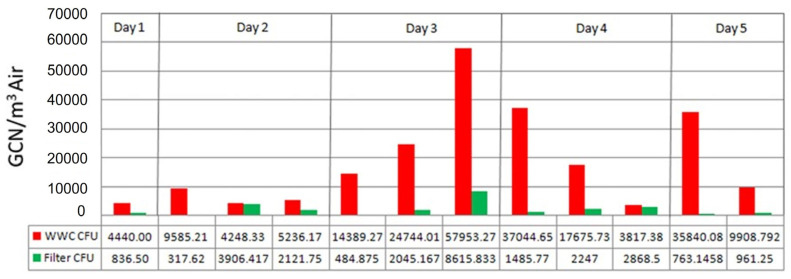
Comparison of the bacterial counts using whole cell qPCR (based on the 16S universal primers) between the WWC and glass fiber filter.

Based on the collected data, the concentrations of the aerosol bacteria within CAFU Study House A are highly dynamic on a daily/hourly level. Depending on the time and date of the collection, the samples show a range of 4000–60,000 CFU/m^3^ indoor bacterial concentrations. The raised levels presented mid-week were likely caused when workers hosed the floors of the facility (Figure 3, Figure 4 and Figure 5).

The reference samples collected at the entrance of the House B also contained high numbers of bioaerosols, albeit about a magnitude less bacteria compared to the indoor samples from Study House A (Figure 4). This is likely due to contamination from Study House A, where the drum fans and the open walls allowed the bioparticles to spread across the 20 m wide area that separates the two structures (Figure 1). There was a significant difference between the concentration of House A and House B aerosols and between House A samples collected using WWC as compared to the Reference Sampler using filters. The *p*-values were 0.01 and 0.003, respectively.

The results of FAME analysis identified the six different bacterial species showing significant fluctuations in the concentrations. The FAME analysis identified the six main isolates as *Bacillus megaterium, Bacillus psychrosaccharolyticus, Staphylococcus hominis, Burkholderia cepacia* GC subgroup B (*Pseudomonas cepacia*), *Bacillus subtilis,* and *Micrococcus luteus*. The highest concentration was found for the *Bacillus* type bacteria.

### 3.2. Bacterial DNA in Filter Samples

At the Study House A, a WWC system was collocated with a standard filter sampler and both systems were set to collect at the same flow rate of 100 L/min. However, the WWC samples yielded approximately an order of magnitude more amplifiable DNA to the PCR system. The contrast in PCR results could be indicative of the high level of viable cells offered by the WWC’s continuous flow operation versus the filter’s significantly lower yield of viable cells. These results are also very similar to the results gathered during the subterranean sampling where a filter sampler was also collocated with a WWC in a harsh environment. It is also important to note that the low performance of the filter may have been assisted by the harsh environment where the moist dust and organic particles collected on the filters could encase the bacteria and help maintain the viability (Figure 5). 

**Figure 6:**
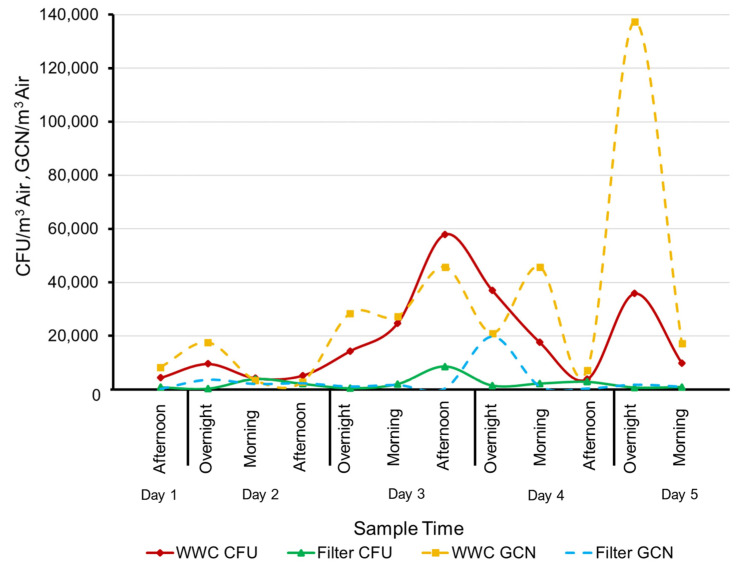
Plating counts for WWC and filter versus the whole cell qPCR (based on the 16S universal primers). PCR data for WWC are erratic likely due to sample preparation steps required to process heavily loaded samples, yet the general trend of the WWC’s PCR data is consistent with plating, and the filter-based PCR data are strongly correlated with plating. The Morning sample was collected at 12:30 PM for a four-h duration, Afternoon sample was collected at 4:30 PM for a four-h duration, and the Overnight sample was collected at 8:30 AM for a sixteen-h duration.

It is important to note that the bacterial count data stated previously in this report are based on plate counts. The 16S Universal Primer based whole-cell qPCR data were not used because of outliers within the WWC dataset. The suspected cause of the outlier data is that the WWC samples required multiple dilution steps because they contained unexpectedly high concentrations of cells and dust. These additional dilution steps cumulatively added error to the analysis and reduced its accuracy. However, the general trend of the WWC’s PCR data is consistent with plating, and the filter-based PCR data are correlated with plating (Figure 6).

### 3.3. Identification of Pathogens

**Figure 7:**
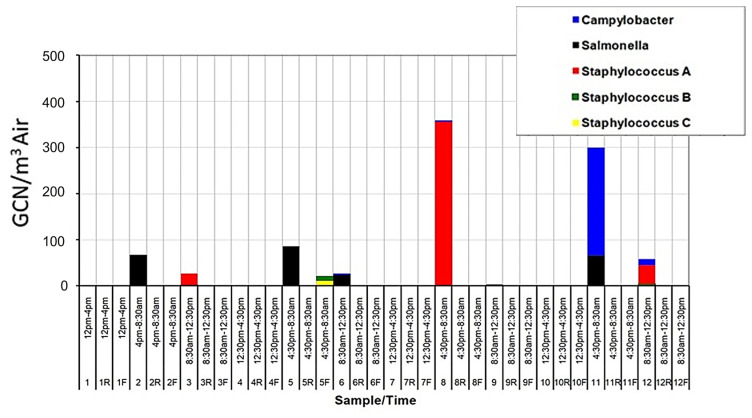
GCN/m^3^ results using microorganism-specific primers. These microorganisms were chosen for their pathogenic and opportunistic nature. Samples 1–12 indicate the WWC collections in House A, 1R–12R the WWC collections in House B and 1F–12F the filter collections in House A.

The results of the PCR analysis, which used primers specific for the pathogens *Campylobacter jejuni, Salmonella enterica,* and *Staphylococcus aureus* (*sea*, *seb* and *sec* sequences), indicated the presence of these organisms in significant concentrations (Figure 7). The highest bacterial concentrations (GCN/m^3^ air), including the potentially pathogenic strains, were found in the overnight samples. This could be due to keeping the animals in a confined space that allows pathogen shedding and spreading, or environmental factors more favorable to these organisms.

### 3.4. Bioaerosol Collection with the WWC in the CAFU Environment

**Figure 8:**
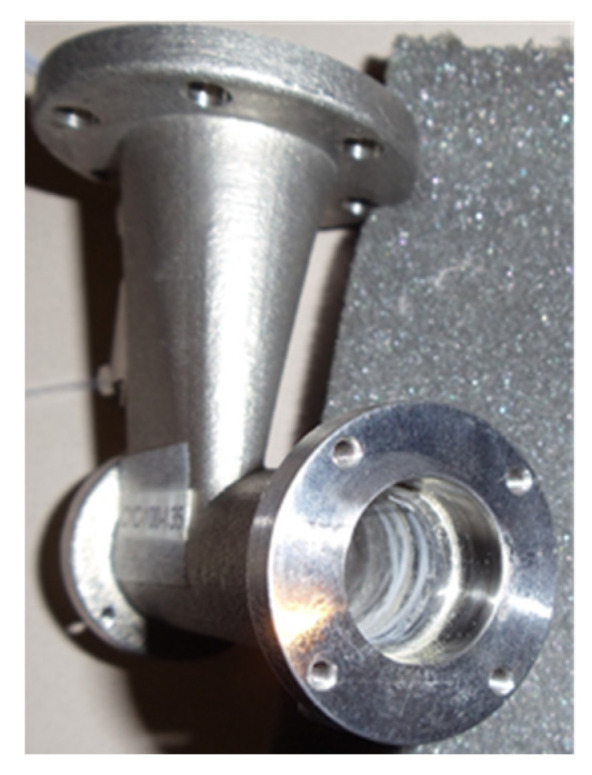
Dust accumulated inside the cyclone, behind the skimmer region. The dust particles deposited by the path of the swirling collection liquid.

The WWC system that has previously been used to successfully collect bioaerosols in dusty environments sampled air continuously during the 5-day period without any signs of clogging, despite the high dust concentration that was also deposited on the inner walls of the stainless-steel cyclone (Figure 8).

Researchers have enabled the cyclones to handle larger amounts of dust by increasing the aspiration port diameters and replacing the liquid lines with wider tubes. The collectors have also been equipped with silencers to attenuate the blower noise that could affect the behavior of the flock, causing the upset animals to stir up more dust.

## 4. Discussion

The study yielded four key results: Firstly, the concentrations of microorganisms in the Confined Animal Feeding Units (CAFU), specifically in Study House A, exhibited a highly dynamic range, with levels ranging from as low as 4000 CFU/m^3^ to as high as 60,000 CFU/m^3^ when collected using the Wetted Wall Cyclone (WWC) system. Secondly, the WWC samples showed approximately an order of magnitude more bacterial DNA compared to the filter samples, indicating reduced bacterial DNA in the filter samples. Thirdly, the study detected significant concentrations of pathogens with known multi-drug resistance such as *Salmonella, Staphylococcus,* and *Campylobacter* in the poultry facility’s aerosol samples. This information is useful and can be applied to inform poultry facilities of efficient cleaning procedures, specifically decontaminating surfaces more often and in between shifts of workers to ensure that less pathogens, which are known to cause infection, become resuspended in the air. Lastly, the WWC system demonstrated effective functionality in the challenging sampling environment of the CAFU. This study will be built upon to sample upwind and downwind locations and analyze the difference in microbiomes.

## Data Availability

The data presented in this study are available from the corresponding author on request.
